# Novel Applications of Accelerometry Data for Health Outcomes in Older Adults: Thinking Beyond MVPA

**DOI:** 10.1093/geroni/igab046.1297

**Published:** 2021-12-17

**Authors:** Jennifer Schrack, Jacek Urbanek, Manini Manini

## Abstract

Physical activity is a well-established predictor of health and longevity. Wearable accelerometers produce high-frequency, time series data that capture multiple aspects of daily physical activity across the spectrum of intensity. Historically, the majority of accelerometry-based physical activity research has employed summary threshold metrics such as moderate-to-vigorous physical activity, or “MVPA.” Although these measures are important for understanding compliance with physical activity guidelines, they underutilize the potential of this data. To advance the science of physical activity in older adults, more sensitive, clinically translatable measures are needed. This symposium will examine the associations between novel measures of accelerometry-derived physical activity and various aging-related health outcomes. Dr. Wanigatunga will discuss the association of physical activity volume and fragmentation with the frailty phenotype in the Study to Understand Vitamin D and Fall Reduction in You (STURDY). Dr. Cai will present evidence on the association of physical activity quantities and patterns with measures of visual impairment in the Baltimore Longitudinal Study of Aging. Ms. Qiao will present a novel accelerometry-derived measure of performance fatigability in the Developmental Epidemiologic Cohort Study. Finally, Dr. Urbanek will discuss the role of accelerometry-derived free-living gait cadence in defining fall risk in STURDY. Collectively, these presentations highlight critical associations between objective measures of physical activity and health outcomes in older adults and illuminate the need for thinking beyond MVPA to improve prevention and intervention efforts.

